# A Novel Approach to Assess the Potency of Topical Corticosteroids

**DOI:** 10.3390/pharmaceutics13091456

**Published:** 2021-09-13

**Authors:** Michael Zvidzayi, Seeprarani Rath, Charles Bon, Sagaran Abboo, Isadore Kanfer

**Affiliations:** 1 Biopharmaceutics Research Institute, Rhodes University, Grahamstown 6139, South Africa; mzvidzayi@gmail.com (M.Z.); s.rath@ru.ac.za (S.R.); 2 Biostudy Solutions LLC, Wilmington, NC 28401, USA; chuck@biostudysolutions.com; 3 Faculty of Pharmacy, Rhodes University, Grahamstown 6139, South Africa; s.abboo@ru.ac.za; 4 Leslie Dan College of Pharmacy, University of Toronto, Toronto, ON M5S 3M2, Canada

**Keywords:** vasoconstrictor assay, skin blanching response, chromameter, topical corticosteroids, active pharmaceutical ingredients, potency, *E_max_* model

## Abstract

The potencies of topical corticosteroid products have mainly been classified using clinical data but in some instances, the US Food and Drug Administration’s (FDA’s) vasoconstrictor assay (VCA) to assess the skin blanching response has also been used. However, the reported skin blanching response data were often based on a single visual reading and lack information on the dose (amount/quantity) or dose duration. Although several lists classifying potencies of various topical corticosteroid products have been published, the inherent potencies of topical corticosteroid raw materials used as active pharmaceutical ingredients (APIs) have not been investigated. The objective was to rank the inherent potencies of topical corticosteroid APIs and to standardize dosing such that the relevant compounds could be compared on a normalized molar basis. The potencies of clobetasol propionate, halcinonide, mometasone furoate, and fluocinolone acetonide were compared using the resulting *E_max_* data following the fitting of the relevant response data to the *E_max_* model where mometasone furoate > fluocinolone acetonide = clobetasol propionate > halcinonide. This ranking lists the respective inherent potencies of the APIs, which will facilitate the choice of a suitable candidate for incorporation into an appropriate topical corticosteroid product for a specific clinical indication.

## 1. Introduction

Unlike other therapeutic classes of drugs, such as antifungals or antibacterials, topical corticosteroid products have been ranked and classified depending on their potencies [1]. The main method to evaluate the potencies of topical corticosteroid products has been through the results of their clinical use and randomized clinical comparative studies [2]. In the USA, topical corticosteroid products are ranked into seven classes from superpotent to least potent while a four-category system is used in Northern Europe, United Kingdom (UK), France, Germany, Netherlands, and New Zealand to classify potencies [1,3,4,5,6]. In New Zealand, class I is the most potent and class IV the least potent, while in continental Europe class I is mildly potent and class IV very highly potent which is confusing and can be easily misinterpreted depending on where the topical corticosteroid product is manufactured and marketed [3,4]. However, the four-category system may not provide adequate discrimination to differentiate significant therapeutic differences among various corticosteroid preparations and between classes [4]. Ideally, topical corticosteroid products in the same group should have similar efficacy and also a similar potential to provoke similar side effects [7].

Whereas several lists describing and classifying the potencies of topical corticosteroid products have been published [1,3,4,6], potency classification of topical corticosteroid raw materials used as active pharmaceutical ingredients (APIs) is lacking.

While the potencies of topical corticosteroid products have mainly been based on the results of their clinical use and randomized clinical comparative studies [2,5,8,9], the vasoconstrictor assay (VCA) has also been used in some instances [1,7,10,11,12,13]. However, when the VCA was used to rank topical corticosteroid products, the literature is conspicuously absent of information relating to the dose or dose duration, and when such information was mentioned, the approach used was generally non-standardized and usually differed from study to study. Furthermore, visual assessments of the skin blanching response were made at various times [2,10,14,15,16,17,18,19,20,21,22] after removal and sometimes the visual 4-point assessment scale was used [2,10,14,15,16,18,23] whereas, at other times, the results were simply recorded as “yes” for a skin blanching response or “no” when skin blanching was not observed [19,21,24]. Furthermore, the concentrations of the relevant topical corticosteroids in a product were not taken into consideration, thus ignoring the molecular weight differences between compounds. For example, 0.1% mometasone furoate creams (Elocon^®^, Ecural^®^) is a purported high potency class but classified as being less potent than 0.5% triamcinolone acetonide cream (Aristocort-HP^®^) which is classified in the lower potency category [6]. The classification of these two formulations does not take into consideration the differences in corticosteroid concentrations used in each formulation but are simply compared on the same scale. Thus, Aristocort-HP^®^ cream may be considered more potent than Elocon^®^ and Ecural^®^ creams only due to its higher molar concentration. Furthermore, discrepancies in the classification of topical corticosteroid product potency also exist in the published literature [10,16,25,26]. Table S1 depicting examples of discrepancies has been provided as supplementary material.

Further, some of the published literature indicates that the VCA without clinical data was used to rank the potencies of topical corticosteroid products giving no weight to clinical outcome, safety, or cost [1,7,11,12,13] while other publications indicate that the VCA with clinical data was used to rank the potencies [5,8,9]. Omission of such critical information on how the potency determinations were made raises concerns about the reliability of the current classifications. Whereas the application of VCA usually requires the use of a chromameter, all rankings and classifications using the VCA were based solely on visual assessment. Based on current knowledge and the use of new reliable, accurate, and innovative technology, the resulting current classifications obtained from visual assessment and non-standardized methodology are thus in need of reassessment using chromametric measurements of the skin blanching response [1,12].

The objective of this study is therefore to rank the potencies of topical corticosteroid APIs using the Food and Drug Administration’s (FDA’s) VCA and to standardize dosing such that the relevant compounds could be compared on a normalized molar basis. This is in distinct contrast to existing classifications where comparisons of potencies take no account of differences in topical corticosteroid concentrations in such products. Although it is well understood that formulation and associated vehicle effects can influence the potency of APIs [27,28], a list indicating the inherent potencies of topical corticosteroids will provide useful information regarding the choice of a specific API for investigation and inclusion in an appropriate topical corticosteroid product for clinical use.

### Correlation of ED_*50*_ and E_max_ with Potency Classification

In a published study [29] using VCA to assess the potencies of some topical corticosteroid products, it was shown that the *ED*_50_ of a stronger potency class product was higher than that of a weaker potency class one. The study was done using 0.05% betamethasone butyrate propionate cream, a purported potency class II corticosteroid, and 0.01% hydrocortisone butyrate, a purported potency class III corticosteroid to determine their respective *ED*_50_ and *E_max_* values. The results revealed that the *ED*_50_ of 0.05% betamethasone butyrate propionate cream (higher potency class) was almost 50% higher than that of 0.01% hydrocortisone butyrate (lower potency class) whereas the respective *E_max_* values were somewhat similar but in the correct rank order, 0.05% betamethasone butyrate propionate cream = 89 and 0.01% hydrocortisone butyrate = 72. However, it has been shown that the *ED*_50_ values were inversely proportional to the potency of the drug using a nonlinear mixed effects model (NONMEM) analysis [30]. This was based on the premise that the *ED*_50_ value relates to the dose duration corresponding to half-maximal response, and as such, to the speed of absorption of a medication. However, the absorption velocity of medication is strongly influenced by several factors including, release capability of the formulation, degree of penetration into the skin, stability of the drug, and potency among others. Hence, the *ED*_50_ value does not only reflect the potency of the product but is clearly influenced by additional factors [30]. These findings suggest that *E_max_* may be the more appropriate parameter that correlates with potency and not the *ED*_50_.

## 2. Materials and Methods

### 2.1. Materials

Four topical corticosteroids were investigated in these studies. The APIs were chosen based on their availability. Clobetasol propionate, mometasone furoate, and fluocinolone acetonide were purchased from Sigma^®^ Aldrich (Kappelweg, Schnelldorf, Germany) and halcinonide was obtained as a gift from Ranbaxy^®^, (Mumbai, India). The solutions were freshly prepared immediately before use in each study and kept away from direct sunlight and at ambient room temperature not exceeding 25 °C. A standard molar concentration (0.0025 M) was chosen for all the respective topical corticosteroids. A 0.0025 M concentration was used to approximate a 0.1% topical corticosteroid solution for each compound. Based on solubility of the APIs in various solvents, analytical grade propylene glycol (MINEMA Chemicals (Pty) Ltd., Clockwork Road, Gauteng, South Africa) was chosen as the appropriate solvent to obtain a standard concentration of 0.0025 M for each of the APIs used. All the APIs were readily soluble in propylene glycol following sonication.

### 2.2. Experimental Design

Ethical approval (Ref No: 160614243, 1 July 2016) was obtained from Pharma-Ethics (Pty) Ltd. research ethics committee (Lyttelton Manor, South Africa), in compliance with the 1964 Declaration of Helsinki and its subsequent amendments, and the study was conducted in the Biopharmaceutics Research Institute Clinic at Rhodes University. Informed consent for screening was obtained from healthy human volunteers between 18 and 35 years of age, and the subjects were subsequently screened. All subjects underwent a pre-study medical examination and only those who complied with all the appropriate inclusion and exclusion criteria were accepted for enrolment into the studies. Subjects who showed an acceptable blanching response and complied with all inclusion and exclusion criteria in compliance with the FDA’s VCA guidance [31] were admitted into the study. Check-in was done the evening before the study day and the participating subjects were confined to the clinic until the end of the study. During check-in, female subjects were required to undergo a pregnancy test. Each subject was also required to undergo an alcohol test and vital signs check which included blood pressure, pulse rate, and body temperature. A medical questionnaire was completed by each trial subject under the direction of the study physician. Dosing of each subject took place in the morning of the study day. A five microliter (5 µL) dose was applied to demarcated sites on the flexor surface of the skin of each subject and left on for the relevant dose duration (i.e., 5, 10, 20, 40, 60, 90, and 150 min). The API solutions were removed after the relevant dose durations from the application sites using cotton swabs, 3 wet wipes (warm water) followed by 2 dry wipes. Chromameter (Model CR 400, Minolta^®^, Osaka, Japan) readings at all sites were taken over 24 h by a single chromameter operator. The chromameter uses tristimulus colorimetry and functions by emitting a white light (using a pulsed xenon arc lamp) onto the application site on the skin and the intensity of reflected light is measured through three particular wavelength filters, (450, 560, and 600 nm). The detected signal is converted into three coordinates: L∗ (luminosity), a∗ (the amount of green or red), and b∗ (the amount of yellow or blue). In accordance with the FDA guidance, only the a-scale data are used in the statistical analysis [32,33,34]. Since clobetasol propionate was used as a comparator in each of the three separate study groups, fresh solutions of that topical corticosteroid were made for each group dosing, i.e., clobetasol propionate (1), clobetasol propionate (2), and clobetasol propionate (3).

### 2.3. Statistical Analysis

Data fitting analyses were carried out using P-Pharm (Simed) software to determine the *E_max_* and *ED*_50_ values where the no-intercept *E_max_* model was used to fit the area under the effect curve (*AUEC*) for each dose duration as stated in the FDA guidance [31]. The data sets for each of the APIs were analyzed with the log-normal distribution assumption for *ED*_50_ to fit the *E_max_* model [31] according to Equation (1) that describes some measure of the elicited effect (*E*) in terms of a baseline effect (*E_0_*), a maximal effect (*E_max_*), and a dose (*D*) at which the effect is half-maximal (*ED*_50_).

(1) $$E=\left(E_{max}\times D\right)/\left(ED_{50}+D\right)$$

where

*E* = Pharmacodynamic effect metric i.e., *AUEC*;

*D* = Duration of exposure (min) to the topical corticosteroid;

*E_max_* = Maximum possible value for “*E*”;

*ED*_50_ = Dose duration necessary to achieve 50% of the *E_max_* response.

The individual a-scale readings were plotted against the reading times over a period of 24 h and the respective areas under the effect curve (*AUEC_0–24_*) values were calculated by the linear trapezoidal method from the baseline adjusted [35] and untreated site corrected a-scale values for each subject at each dose duration for the respective APIs. These *AUEC_0–24_* values were plotted against the respective dose durations and used to estimate the *E_max_* model that best described (minimum Akaike information criterion-*AIC*) the data for the APIs.

The one-way analysis of variance (ANOVA) was used to determine the presence/absence of significant differences in the *E_max_* parameters among the different topical corticosteroids and was carried out using SAS^®^ R statistical software (SAS Institute Inc., Cary, NC, USA).

## 3. Results

### 3.1. Study 1: Halcinonide vs. Clobetasol Propionate (1)

**Figure 1:**
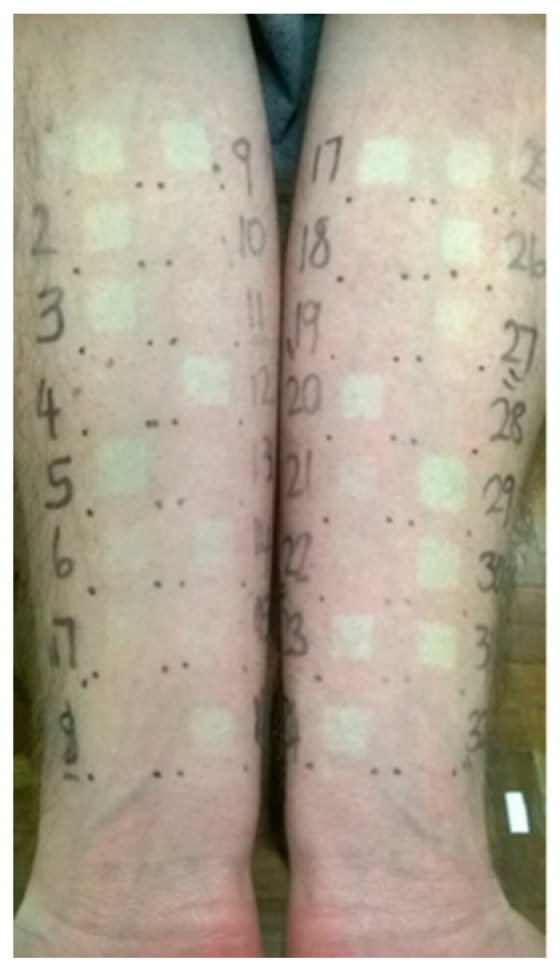
Typical blanching responses after randomized application of 0.0025 M solutions of halcinonide and clobetasol propionate (1).

Ten subjects, (2 females and 8 males) who were Caucasians completed the study and the chromameter response data of all 10 subjects were obtained for each of the 0.0025 M halcinonide and clobetasol propionate (1) solutions. No adverse drug reactions or other clinical events were encountered during this study. Figure 1 illustrates the typical blanching responses after randomized application of 0.0025 M solutions of halcinonide and clobetasol propionate (1). Details of the halcinonide and clobetasol propionate (1) responses obtained at various dose durations are provided under supplementary material in Table S2.

**Figure 2:**
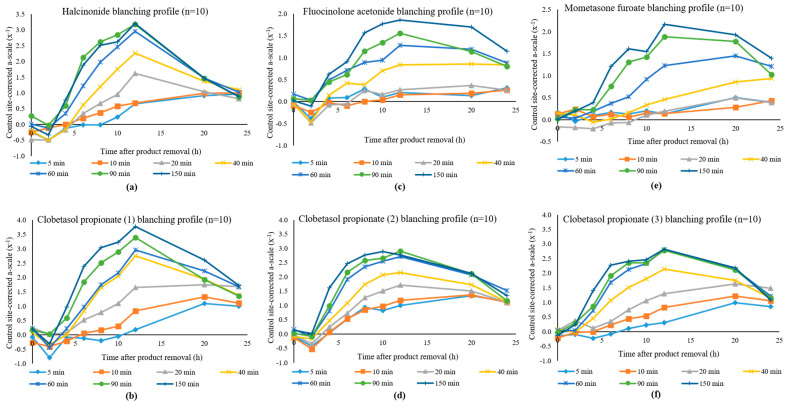
Blanching profiles for (**a**) halcinonide; (**b**) clobetasol propionate (1); (**c**) fluocinolone acetonide; (**d**) clobetasol propionate (2); (**e**) mometasone furoate; (**f**) clobetasol propionate (3).

After the various times of exposure (i.e., at 5, 10, 20, 40, 60, 90, and 150 min dose durations), the blanching effect of both halcinonide and clobetasol propionate (1) peaked at 12 h after product removal, decreasing thereafter. The baseline adjusted and untreated site corrected a-scale values for halcinonide and clobetasol propionate (1) were plotted against time after product removal illustrating the mean blanching responses of the ten subjects as shown in Figure 2a,b, respectively. Since negative values are obtained, these are plotted as x^−1^ on the *y*-axis. The plots show that as the dose durations increase, there is a corresponding increase in the skin blanching response.

**Figure 3:**
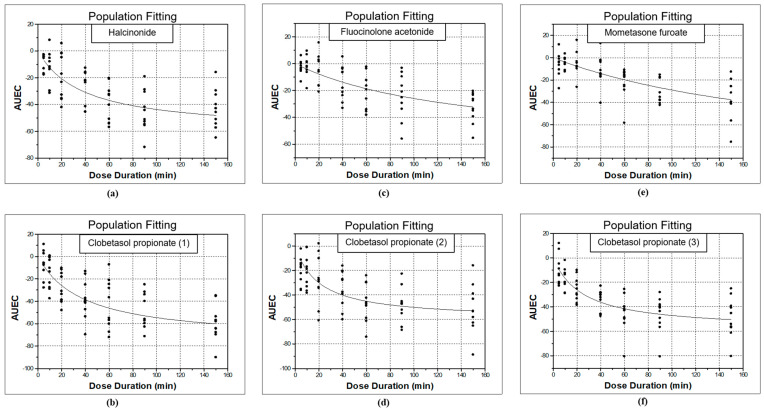
Fitted *E_max_* model of *AUEC* data derived from the chromameter a-scale values for (**a**) halcinonide; (**b**) clobetasol propionate (1); (**c**) fluocinolone acetonide; (**d**) clobetasol propionate (2); (**e**) mometasone furoate; (**f**) clobetasol propionate (3).

The data sets were analyzed as previously described and the respective *AUEC* values were calculated. The *AUEC* values were used to estimate the *E_max_* model that best described the data for halcinonide and clobetasol propionate (1) as shown in Figure 3a,b, respectively.

### 3.2. Study 2: Fluocinolone Acetonide vs. Clobetasol Propionate (2)

Ten subjects, (three females and seven males) who were Caucasians completed this study, and no adverse drug reactions or other clinical events were encountered. As in the case of the APIs in Study 1, the blanching responses peaked at 12 h after product removal, decreasing thereafter. The baseline corrected and untreated site corrected a-scale values for fluocinolone acetonide and clobetasol propionate (2) were plotted against the time after product removal illustrating the mean blanching responses of the ten subjects as shown in Figure 2c,d, respectively. As in previous observations, the plots show that as the dose durations increase, there is a corresponding increase in the skin blanching response.

The *AUEC* values were calculated as previously described and used to estimate the *E_max_* model that best described (minimum *AIC*) the data for fluocinolone acetonide and clobetasol propionate (2) as shown in Figure 3c,d, respectively.

### 3.3. Study 3: Mometasone Furoate vs. Clobetasol Propionate (3)

Ten subjects, (4 females and 6 males) who were Caucasians completed this study, and no adverse drug reactions or other clinical events were encountered during the study. As in the previous groups and comparisons, the blanching effect of both mometasone furoate and clobetasol propionate (3) peaked at 12 h after product removal, decreasing thereafter. The baseline corrected and untreated site corrected a-scale values for mometasone furoate and clobetasol propionate (3) were plotted against the time after product removal illustrating the mean blanching responses of the ten subjects as shown in Figure 2e,f, respectively. The plots show that as the dose durations increase, there is a corresponding increase in the skin blanching response.

The data sets were analyzed as previously described and the respective *AUEC* values were calculated. The *AUEC* values were used to estimate the *E_max_* model that best described (minimum *AIC*) the data for mometasone furoate and clobetasol propionate (3) as shown in Figure 3e,f, respectively.

### 3.4. Comparisons of Topical Corticosteroids

Each study consisted of a pair of topical corticosteroids where clobetasol propionate was included in each as a comparator. Table 1 provides a summary of the respective *ED*_50_, *E_max_*, and *AIC* values.

In order to normalize the *E_max_* model parameters for clobetasol propionate in the three studies, a correction factor was calculated by subtracting the mean clobetasol propionate *E_max_* values in each study from the mean clobetasol propionate *E_max_* obtained in Study 1. These correction factors, which were used to normalize the results for the three studies were as follows: Study 1 = 0, Study 2 = −15.39 and Study 3 = −16.64. Hence, the individual subject data from Study 1 (Table 2) do not include a normalization column in the table and all results were normalized to the mean for clobetasol propionate in Study 1 where the individual *E_max_* values obtained in the modelling for that study could be used without any correction.

The *ED*_50_ and *E_max_* values for each individual subject included in the population *E_max_* modelling in all three studies were obtained from P-Pharm. Table 2, Table 3 and Table 4 list these *E_max_* values for all the subjects (*n* = 30) from Study 1, Study 2, and Study 3 respectively. The arithmetic mean of the individual *E_max_* values is equal to the population mean (parameter estimate) for the model. Under the log-normal distribution assumption for *ED*_50_, the geometric mean of the individual subject *ED*_50_ values equals the population *ED*_50_ estimate for the model. The normalized *E_max_* values obtained by subtracting the respective correction factors from the calculated values are shown in Table 3 and Table 4.

#### 3.4.1. ANOVA Comparisons

Out of the 30 subjects who were included in the potency studies, 21 were male and 9 were female. The *E_max_* values of 30 subjects (3 studies × 10 subjects/study) for clobetasol propionate and of 10 subjects each for halcinonide, fluocinolone acetonide, and mometasone furoate were statistically evaluated using a one-way ANOVA [36] to test if the *E_max_* means from the different topical corticosteroids were equal. The results indicated that the overall F test was statistically significant (F = 46.07; *p* < 0.0001). As the model *p*-value was less than 0.05, the null hypothesis that the *E_max_* values for all topical corticosteroids are equal was rejected. The alternative hypothesis that the *E_max_* of at least one topical corticosteroid differed from that of at least one other topical corticosteroid was accepted.

#### 3.4.2. Pairwise Comparisons

Tukey’s studentized range-honestly significant difference (HSD) Test was used for the multiple pairwise comparisons to determine which, if any, topical corticosteroid *E_max_* means differed significantly from any of the other means. Table 5 shows the resulting *E_max_* comparisons. The topical corticosteroid mean *E_max_* values with the same Tukey grouping letter (A, B, or C) were not detected as being statistically different from each other. Those with different Tukey grouping letters were statistically different from each other.

The mean *E_max_* value for mometasone furoate was statistically different from those of clobetasol propionate, fluocinolone acetonide, and halcinonide. While both clobetasol propionate and fluocinolone acetonide have statistically equivalent mean *E_max_* values, both of these topical corticosteroids differ statistically from halcinonide and mometasone furoate.

## 4. Discussion

### Potency Rankings of Topical Corticosteroids Based on E_max_ Values

The molecular weights (MWs) of clobetasol propionate (MW 467.0 g/mol), halcinonide (MW 455.0 g/mol), and fluocinolone acetonide (MW 452.5 g/mol), except for mometasone furoate (MW 521.4 g/mol) were very similar. Moreover, the log P values for mometasone furoate (log P 3.9), clobetasol propionate (log P 3.8), and halcinonide (log P 3.6) were similar, except for fluocinolone acetonide (log P 2.5) [37]. Although any possible effects on permeation due to MW and log P may affect the blanching responses, the correlation of these specific physicochemical properties with the dose response results will require further investigation. Since the APIs were dissolved in the same vehicle i.e., propylene glycol, the results obtained provide an appropriate comparative inherent potency ranking for the respective APIs.

Based on the *E_max_* values as the main criterion for potency classification as previously described, the following potency ranking of the relevant topical corticosteroids is shown below, viz.:

***mometasone furoate > fluocinolone acetonide = clobetasol propionate > halcinonide***

To categorize these compounds, a classification system based on *E_max_* data is hereby proposed:

Mometasone furoate is assigned to a tentative class I (highly potent) based on its *E_max_*, with fluocinolone acetonide and clobetasol propionate as tentative class II (potent) since they were found to be equipotent but less potent than mometasone furoate. Consequently, halcinonide is assigned as tentative class III (mildly potent), as it was less potent than the other topical corticosteroids evaluated. When more data become available relating to the *E_max_* values of topical corticosteroids in future studies, it is possible that some of those topical corticosteroids may be associated with even higher (i.e., more negative) *E_max_* values than mometasone furoate and similarly, some topical corticosteroids may have *E_max_* values between the most potent (mometasone furoate) and the least potent (halcinonide) in these studies. If a more potent topical corticosteroid is identified, mometasone furoate may need to be relegated to class II and the current class II and class III compounds either may remain in their respective classes or even be relegated to lower classes (low potency). Furthermore, it is also quite probable that some other topical corticosteroids may have *E_max_* values between those currently found in these studies and would thus need to be accommodated in the classification list too. Any topical corticosteroid can thus be included based on their *E_max_* values and a new Roman numeral class can be added when necessary with a concomitant shifting of all or some of the previously designated topical corticosteroids. All potency classes and the classifications should be based on the current normalized statistical comparisons for significant differences between *E_max_* values and cut-off values of *E_max_* will need to be established to separate the various classes.

Interestingly, since clobetasol propionate-containing formulations have been considered to be the most potent of topical corticosteroid formulations [3,4,6], one would assume that clobetasol propionate itself should also be the most potent. However, when appropriate standardized methods such as those described in this paper are used, it was shown that clobetasol propionate itself, is in fact, less potent than mometasone furoate. Clobetasol propionate was also expected to be more potent than fluocinolone acetonide and halcinonide, but it was shown that fluocinolone acetonide is similar in potency based on its *E_max_* value. These results reiterate the importance of having an appropriate potency classification for topical corticosteroid compounds as classification of these may very well differ from the published potency classifications of topical corticosteroid products.

## 5. Conclusions

The potencies of four topical corticosteroid APIs were assessed using the VCA on a normalized molar basis. A potency classification system based on *E_max_* data is proposed where the results indicate that mometasone furoate > fluocinolone acetonide = clobetasol propionate > halcinonide. The clinical choice of topical corticosteroid products is governed by the type and severity of inflammatory skin condition/lesions, location of the lesion, age of the patient, etc. For example, dermatologists recommend lower potency agents for infants and the elderly owing to an increased surface-to-weight ratio and skin fragility, respectively. Agents belonging to the lower potency classes are used to treat acute inflammatory lesions of the face and other body parts with thinner skin, whereas highly potent agents are preferred in the treatment of chronic, keratotic, or lichenified lesions found on surfaces with thicker skin e.g., palms, soles. Additionally, the type of lesion to be treated influences the choice of vehicle, e.g., ointment bases are recommended for lichenified lesions as they improve drug penetration due to occlusive effect and subsequent hydration [6]. Using the VCA and the resulting *E_max_* data to rank the inherent potencies of individual APIs will provide useful information that should aid future development and optimization of topical corticosteroid products to facilitate the choice of a suitable API to provide optimum safety and efficacy and allow the incorporation of an appropriate strength for a specific clinical indication. This approach can be readily applied as a standard method to determine the *E_max_* value as an indication of the potency of a specific topical corticosteroid product according to a compiled classification list containing such data and also can be used to directly compare potency between products in a comparative study.

## Figures and Tables

**Table 1:** Summary of population model results.

Study	Topical Corticosteroid	*ED*_50_	*E_max_*	*AIC*
1	Clobetasol propionate (1)	38.10	−75.44 ± 12.89	3.93
2	Clobetasol propionate (2)	19.83	−60.05 ± 13.31	3.79
3	Clobetasol propionate (3)	24.83	−58.79 ± 15.65	3.57
1	Halcinonide	38.06	−60.31 ± 3.75	3.84
2	Fluocinolone acetonide	142.46	−62.84 ± 5.98	3.64
3	Mometasone furoate	225.77	−94.45 ± 0.21	3.73

**Table 2:** Individual subject results (*E_max_* values) from Study 1.

Subject	Clobetasol Propionate (1)	Halcinonide
1	−80.66	−66.45
2	−66.29	−57.89
3	−91.31	−59.61
4	−68.67	−56.39
5	−74.33	−60.27
6	−50.49	−56.90
7	−93.04	−63.16
8	−83.35	−55.28
9	−66.84	−63.06
10	−79.41	−64.10

**Table 3:** Individual subject results from Study 2.

Subject	Observed *E_max_* Values		Normalized *E_max_* Values	
	Clobetasol Propionate (2)	Fluocinolone Acetonide	Clobetasol Propionate (2)	Fluocinolone Acetonide
1	−56.98	−59.20	−72.37	−74.59
2	−88.12	−66.48	−103.51	−81.87
3	−39.75	−58.76	−55.13	−74.15
4	−68.04	−63.66	−83.43	−79.05
5	−62.63	−59.23	−78.02	−74.62
6	−56.50	−55.39	−71.89	−70.78
7	−68.51	−73.88	−83.90	−89.27
8	−58.31	−59.44	−73.70	−74.83
9	−56.13	−71.39	−71.52	−86.78
10	−45.52	−60.98	−60.91	−76.37

**Table 4:** Individual subject results from Study 3.

Subject	Observed *E_max_* Values		Normalized *E_max_* Values	
	Clobetasol Propionate (3)	Mometasone Furoate	Clobetasol Propionate (3)	Mometasone Furoate
1	−69.72	−94.78	−86.36	−111.43
2	−61.19	−94.50	−77.83	−111.15
3	−35.84	−94.30	−52.48	−110.95
4	−54.75	−94.23	−71.39	−110.87
5	−93.29	−94.83	−109.93	−111.47
6	−49.71	−94.27	−66.36	−110.91
7	−65.43	−94.48	−82.07	−111.13
8	−44.62	−94.25	−61.27	−110.89
9	−54.50	−94.36	−71.15	−111.00
10	−58.90	−94.48	−75.54	−111.12

**Table 5:** Pairwise *E_max_* comparisons.

Topical Corticosteroid	N	Mean (*E_max_*)	Tukey Grouping ^1^
Halcinonide	10	−60.31 ± 3.75	A
Clobetasol propionate	30	−75.44 ± 13.51	B
Fluocinolone acetonide	10	−78.23 ± 5.98	B
Mometasone furoate	10	−111.09 ± 0.21	C

^1^ Topical corticosteroid means (*E_max_*) with the same letter are not significantly different.

## Data Availability

Data will be provided on request.

## Supplementary Materials

The following are available online at https://www.mdpi.com/article/10.3390/pharmaceutics13091456/s1, Table S1: Types of discrepancies observed in potency ranking of topical corticosteroids, Table S2: Application template showing details of the halcinonide and clobetasol propionate (1) responses obtained at various dose durations in Figure 1.

## References

[1] Humbert P., Guichard A. (2015). The topical corticosteroid classification called into question: Towards a new approach. Exp. Dermatol..

[2] Cornell R.C., Stoughton R.B. (1985). Correlation of the vasoconstriction assay and clinical activity in psoriasis. Arch. Dermatol..

[3] Topical Steroid. https://dermnetnz.org/topics/topical-steroid/.

[4] Green C., Colquitt J.L., Kirby J., Davidson P., Payne E. (2004). Clinical and cost-effectiveness of once-daily versus more frequent use of same potency topical corticosteroids for atopic eczema: A systematic review and economic evaluation. Health Technol. Assess..

[5] Fusaro R.M. (1988). Flexible classification for the clinical potency of topical corticosteroid proprietaries. Drug Intell. Clin. Pharm..

[6] Hengge U.R., Ruzicka T., Schwartz R.A., Cork M.J. (2006). Adverse effects of topical glucocorticosteroids. J. Am. Acad. Dermatol..

[7] Wiedersberg S., Leopold C.S., Guy R.H. (2008). Bioavailability and bioequivalence of topical glucocorticoids. Eur. J. Pharm. Biopharm..

[8] Camisa C., Camisa C. (2004). Corticosteroids. Handbook of Psoriasis.

[9] Stoughton R.B., Tisi R., Ranney H. (1982). Principles of topical steroid treatment in skin diseases. Clinical Topics in Internal Medicine.

[10] Cornell R.C. (1992). Clinical trials of topical corticosteroids in psoriasis: Correlations with the vasoconstrictor assay. Int. J. Dermatol..

[11] Hepburn D.J., Aeling J.L., Weston W.L. (1996). A reappraisal of topical steroid potency. Pediatr. Dermatol..

[12] Burkholder B. (2000). Topical corticosteroids: An update. Curr. Probl. Dermatol..

[13] Ference J.D., Last A.R. (2009). Choosing topical corticosteroids. Am. Fam. Physician.

[14] McKenzie A.W., Atkinson R.M. (1964). Topical Activities of Betamethasone Esters in Man. Arch. Dermatol..

[15] Jackson D.B., Thompson C., McCormack J.R., Guin J.D. (1989). Bioequivalence (bioavailability) of generic topical corticosteroids. J. Am. Acad. Dermatol..

[16] Caron D., Queille-Roussel C., Shah V.P., Schaefer H. (1990). Correlation between the drug penetration and the blanching effect of topically applied hydrocortisone creams in human beings. J. Am. Acad. Dermatol..

[17] Stoughton R.B., Wullich K. (1989). The same glucocorticoid in brand-name products. Does increasing the concentration result in greater topical biologic activity?. Arch. Dermatol..

[18] Child K.J., English A.F., Gilbert H.G., Hewitt A., Woollett E.A. (1968). Vasoconstrictor and systemic activities of topical steroids. Arch. Dermatol..

[19] Stoughton R.B. (1972). Bioassay system for formulations of topically applied glucocorticosteroids. Arch. Dermatol..

[20] McKenzie A.W. (1962). Percutaneous Absorption of Steroids. Arch. Dermatol..

[21] Place V.A., Velazquez J.G., Burdick K.H. (1970). Precise evaluation of topically applied corticosteroid potency. Modification of the Stoughton-McKenzie assay. Arch. Dermatol..

[22] Haigh J.M., Kanfer I., Meyer E., Smith E. (1985). Relative potencies of topical corticosteroid formulations. Br. J. Dermatol..

[23] Coldman M.F., Lockerbie L., Laws E.A. (1971). The evaluation of several topical corticosteroid preparations in the blanching test. Br. J. Dermatol..

[24] McKenzie A.W., Stoughton R.B. (1962). Method for Comparing Percutaneous Absorption of Steroids. Arch. Dermatol..

[25] Allenby C.F., Sparkes C.G. (1981). Halogenation and topical corticosteroids: A comparison between the 17-butyrate esters of hydrocortisone and clobetasone in ointment bases. Br. J. Dermatol..

[26] (2019). Topical Corticosteroids. British National Formulary.

[27] Senyigit T., Ozer O. (2012). Corticosteroids for skin delivery: Challenges and new formulation opportunities. Glucocorticoids-New Recognition of Our Familiar Friend.

[28] Kirkland R., Pearce D.J., Balkrishnan R., Feldman S.R. (2006). Critical factors determining the potency of topical corticosteroids. J. Dermatolog. Treat..

[29] Keida T., Hayashi N., Kawashima M. (2006). Application of the Food and Drug Administration (FDA) bioequivalent guidance of topical dermatological corticosteroid in yellow-skinned Japanese population: Validation study using a chromameter. J. Dermatol..

[30] Singh G.J., Adams W.P., Lesko L.J., Shah V.P., Molzon J.A., Williams R.L., Pershing L.K. (1999). Development of in vivo bioequivalence methodology for dermatologic corticosteroids based on pharmacodynamic modeling. Clin. Pharmacol. Ther..

[31] US Food and Drug Administration (1995). Guidance for Industry: Topical Dermatologic Corticosteroids—In Vivo Bioequivalence.

[32] Kanfer I., Maibach H., Berner B., Gordi T., Benson H., Roberts M. (2021). The Vasoconstrictor Assay (VCA): Then and Now. Drug Delivery Approaches: Perspectives from Pharmacokinetics and Pharmacodynamics.

[33] Kanfer I., Tettey-Amlalo R.N.O., Au W.L., Hughes-Formella B., Shargel L., Kanfer I. (2016). Assessment of topical dosage forms intended for local or regional activity. Generic Drug Product Development: Specialty Dosage Forms.

[34] Kanfer I., Shah V.P., Maibach H.I., Jenner J. (2014). Methods for the Assessment of Bioequivalence of Topical Dosage Forms: Correlations, Optimization Strategies, and Innovative Approaches. Topical Drug Bioavailability, Bioequivalence and Penetration.

[35] Holford N.H., Sheiner L.B. (1981). Understanding the dose-effect relationship: Clinical application of pharmacokinetic-pharmacodynamic models. Clin. Pharmacokinet..

[36] SAS Institute Inc (2004). SAS/STAT 9.1: User’s Guide.

[37] Kim S., Chen J., Cheng T., Gindulyte A., He J., He S., Li Q., Shoemaker B.A., Thiessen P.A., Yu B. (2021). PubChem in 2021: New data content and improved web interfaces. Nucleic Acids Res..

