# Correction: Zvidzayi et al. A Novel Approach to Assess the Potency of Topical Corticosteroids. *Pharmaceutics* 2021, *13*, 1456

**DOI:** 10.3390/pharmaceutics14102006

**Published:** 2022-09-22

**Authors:** Michael Zvidzayi, Seeprarani Rath, Charles Bon, Sagaran Abboo, Isadore Kanfer

**Affiliations:** 1 Biopharmaceutics Research Institute, Rhodes University, Grahamstown 6139, South Africa; 2 Biostudy Solutions LLC, Wilmington, NC 28401, USA; 3 Faculty of Pharmacy, Rhodes University, Grahamstown 6139, South Africa; 4 Leslie Dan College of Pharmacy, University of Toronto, Toronto, ON M5S 3M2, Canada

## 1. Error in Figure

In the original publication [1], there was a mistake in the published *y*-axes labelling in Figure 2. Blanching profiles for (a) halcinonide; (b) clobetasol propionate (1); (c) fluocinolone acetonide; (d) clobetasol propionate (2); (e) mometasone furoate; (f) clobetasol propionate (3)”. The *y*-axes in Figure 2 should be “Control site-corrected a-scale (×−1)”, where (×−1) refers to multiplication by −1 and not “x^−1^ (x to the power −1)”.

The corrected *y*-axes labelling in Figure 2 is as follows and appears below:

## 2. Text Correction

There was an error in the original publication. The sentence “Since negative values are obtained, these are plotted as x^−1^ on the *y*-axis.” has been amended to read as follows: “Since negative values are obtained, these are multiplied by −1 and plotted on the *y*-axis.”

A correction has been made to Section 3.1, Paragraph 2:

After the various times of exposure (i.e., at 5, 10, 20, 40, 60, 90, and 150 min dose durations), the blanching effect of both halcinonide and clobetasol propionate (1) peaked at 12 h after product removal, decreasing thereafter. The baseline adjusted and untreated site corrected a-scale values for halcinonide and clobetasol propionate (1) were plotted against time after product removal illustrating the mean blanching responses of the ten subjects as shown in Figure 2a,b, respectively. Since negative values are obtained, these are multiplied by −1 and plotted on the *y*-axis. The plots show that as the dose durations increase, there is a corresponding increase in the skin blanching response. 

The authors apologize for any inconvenience caused and state that the scientific conclusions are unaffected. This correction was approved by the Academic Editor. The original publication has also been updated.

## Figures and Tables

**Figure 2:**
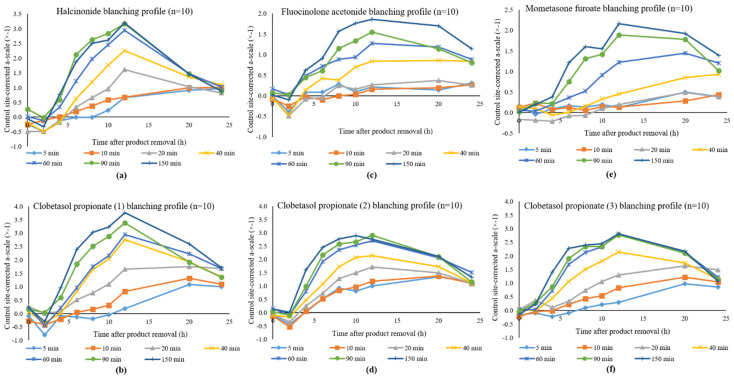
Blanching profiles for (**a**) halcinonide; (**b**) clobetasol propionate (1); (**c**) fluocinolone acetonide; (**d**) clobetasol propionate (2); (**e**) mometasone furoate; (**f**) clobetasol propionate (3).
