# Supplementary material for: A Novel Approach to Assess the Potency of Topical Corticosteroids
Source: Pharmaceutics. 2021 Sep 13;13(9):1456. doi: 10.3390/pharmaceutics13091456 (PMC8466269; doi:10.3390/pharmaceutics13091456)
Supplement: Supplementary file 1 [file pharmaceutics-13-01456-s001.zip › pharmaceutics-1305319 - supplementary(1).pdf]

pharmaceutics

MDPI

Supplementary Materials: A Novel Approach to Assess the Potency of Topical Corticosteroids

Michael Zvidzayi, Seeprarani Rath, Charles Bon, Sagaran Abboo and Isadore Kanfer

Table S1. Types of discrepancies observed in potency ranking of topical corticosteroids.

Types of discrepancies	Description		
Generic and innovator products	Some generic formulations have been shown to be less or more potent than their brand-name equivalent indicating a discrepancy between clinical assessment and VCA [1]. However, the data generated did not comply with the requirements of the VCA guidance since only a simple single point assessment was used.		
Same product under different potency classes	0.1% mometasone furoate (Elocon®)<br>UK - potent (method of assessment not provided) [2]<br>USA - ointment: class II (potent), cream: class IV (midstrength) [3]<br>The Monthly Prescribing Reference (MPR) - cream, ointment and lotion: intermediate potency [4]		
Incomplete API and product information	UK - None of the items indicate the type of formulation and most do not state the percentage API incorporated in the dosage form [2]<br>New Zealand - Apart from betamethasone dipropionate products, no mention is made of the formulation or corticosteroid concentration [5]		
Classification criteria in different countries	The number of classes and order of potency vary between the classification systems used in different countries		
	USA	-	Seven classes (descending order of potency) [3]<br>Class I: superpotent to Class VII: least potent
	Australia	-	Four classes (ascending order of potency) [6]<br>Class I: mild to Class IV: very potent
	Europe	-	Four classes (ascending order of potency) [7]<br>Class I: mildly potent to Class IV: very potent
	New Zealand	-	Four classes (descending order of potency) [5]<br>Class I: very potent/ superpotent to Class IV: mild (potency classification based on a comparison with hydrocortisone)
	UK	-	Four classes (descending order of potency) [2]<br>Class I: very potent to Class IV: mild
Classification based on skin pathology	Eumovate® (0.05% clobetasone 17-butyrate) *vs* Locoid® (0.1% hydrocortisone 17-butyrate) ointments in eczema and psoriasis patients [10]<br>Eczema: Eumovate® = Locoid®<br>Psoriasis: Eumovate® > Locoid®		
Concentration and potency	Potency is not always concentration dependent [8]. In some cases, different concentrations are equipotent but not in others, *viz.*:<br>Kenalog® creams: 0.025% = 0.1% = 0.5%<br>Aristocort® creams: 0.025% = 0.1% = 0.5%<br>Aristocort® ointments: 0.1% = 0.5%<br>Aristocort A® creams: 0.025% = 0.5% < 0.1%<br>Hytone® creams: 1% = 2.5%<br>Synalar® creams: 0.2% > 0.025% > 0.01%<br>Topicort® creams: 0.25% = 0.5%<br>Valisone® creams: 0.1% > 0.01%		

Correlation of clinical studies with VCA data	20 out of 23 comparisons - VCA data in agreement with clinical study [9] although non-validated VCA used.<br>Author stated "it would clearly be advantageous to develop a system for evaluating the potency of these compounds without having to rely on clinical tests"

Table S2. Application template showing details of the halcinonide and clobetasol propionate (1) responses obtained at various dose durations in Figure 1.

Right Arm				Left Arm			
**Site number**	**API-Dose duration**	**Site number**	**API-Dose duration**	**Site number**	**API-Dose duration**	**Site number**	**API-Dose duration**
1	H-150	9	CP-60	17	CP-90	25	H-40
2	CP-40	10	UT	18	UT	26	CP-20
3	H-60	11	H-10	19	H-5	27	H-10
4	UT	12	H-90	20	CP-40	28	UT
5	CP-90	13	CP-20	21	H-20	29	CP-150
6	H-20	14	H-40	22	CP-10	30	H-90
7	CP-10	15	CP-5	23	H-60	31	CP-60
8	H-5	16	CP-150	24	H-150	32	CP-5

H – halcinonide, CP – clobetasol propionate (1), UT – untreated site and 5, 10, 20, 40, 60, 90, 150 – corresponding dose durations in minutes.

References

1. Olsen, E.A. A double-blind controlled comparison of generic and trade-name topical steroids using the vasoconstriction assay. *Arch. Dermatol.* **1991**, *127*, 197–201.
2. Topical corticosteroids. In *British National Formulary*; British Medical Association and Royal Pharmaceutical Society of Great Britain: London, 2019; pp. 1241–1242.
3. Hengge, U.R.; Ruzicka, T.; Schwartz, R.A.; Cork, M.J. Adverse effects of topical glucocorticosteroids. *J. Am. Acad. Dermatol.* **2006**, *54*, 1–15, doi:10.1016/j.jaad.2005.01.010.
4. Topical Steroid Potencies Available online: https://www.empr.com/home/clinical-charts/topical-steroid-potencies/ (accessed on Aug 3, 2021).
5. Topical steroid. Available online: https://dermnetnz.org/topics/topical-steroid/ (accessed on Mar 26, 2021).
6. Mooney, E.; Rademaker, M.; Dailey, R.; Daniel, B.S.; Drummond, C.; Fischer, G.; Foster, R.; Grills, C.; Halbert, A.; Hill, S.; et al. Adverse effects of topical corticosteroids in paediatric eczema: Australasian consensus statement. *Australas. J. Dermatol.* **2015**, *56*, 241–251, doi:https://doi.org/10.1111/ajd.12313.
7. Green, C.; Colquitt, J.; Kirby, J.; Davidson, P.; Payne, E. Clinical and cost-effectiveness of once-daily versus more frequent use of same potency topical corticosteroids for atopic eczema: a systematic review and economic evaluation. *Health Technol. Assess. (Rockv).* **2004**, *8*, iii,iv, 1-120, doi:10.3310/hta8470.
8. Stoughton, R.B.; Wullich, K. The Same Glucocorticoid in Brand-Name Products: Does Increasing the Concentration Result in Greater Topical Biologic Activity? *Arch. Dermatol.* **1989**, *125*, 1509–1511, doi:10.1001/archderm.1989.01670230051007.
9. Cornell, R.C. Clinical trials of topical corticosteroids in psoriasis: correlations with the vasoconstrictor assay. *Int. J. Dermatol.* **1992**, *31 Suppl 1*, 38–40, doi:10.1111/j.1365-4362.1992.tb04012.x.
